# The effect of SLC5A5 gene expression in tumor tissues on refractoriness to radioactive iodine treatment of differentiated thyroid carcinomas

**DOI:** 10.5339/qmj.2025.5

**Published:** 2025-02-05

**Authors:** Aslı Erten, Zeynel Abidin Sayiner, Suna Erkılıç, Sibel Oğuzkan Balcı, Ersin Akarsu

**Affiliations:** ^1^Department of Internal Medicine, Faculty of Medicine, University of Gaziantep, Gaziantep, Turkey; ^2^Department of Endocrinology and Metabolism, Faculty of Medicine, Gaziantep SANKO University, Gaziantep, Turkey; ^3^Department of Pathology, Faculty of Medicine, University of Gaziantep, Gaziantep, Turkey; ^4^Department of Medical Biology, Faculty of Medicine, University of Gaziantep, Gaziantep, Turkey; ^5^Department of Endocrinology and Metabolism, Faculty of Medicine, University of Gaziantep, Gaziantep, Turkey *Correspondence: Zeynel Abidin Sayiner. Email: zeynelasayiner@hotmail.com

**Keywords:** Thyroid neoplasms, radioiodine therapy, solute carrier family 5 member 5, thyroid nodule

## Abstract

**Background and objective:**

The majority of thyroid cancer patients have a good prognosis. Even in advanced disease, the radioactive iodine (RAI) response improves the prognosis. However, RAI refractoriness poses a significant challenge for these patients. The objective of the study was to assess the expression of SCL5A5 as a potential marker for predicting future resistance to radioiodine treatment.

**Materials and methods:**

Radioactive iodine-refractory papillary thyroid carcinoma (RAIR-PTC) and iodine-sensitive papillary thyroid carcinoma (PTC) were included in the study. Demographic and clinicopathological data were retrospectively analyzed. RNA samples were converted to cDNA. Gene expression reactions were performed using synthesized solute carrier family 5 member 5 (SLC5A5) and glyceraldehyde-3-phosphate dehydrogenase (GADPH) primer samples.

**Results:**

Of the patients, 51 (61.4%) had iodine-sensitive PTC and 32 (38.5%) were RAIR-PTC. Patients were followed up for 8 ± 6.4 years. The mean age at diagnosis was higher in the RAIR-PTC group (56.56 ± 15.22 years vs. 46.82 ± 12.43 years, p = 0.002). The PTC group had higher SLC5A5 gene expression than RAIR-PTC. In addition, no statistically significant correlation was observed between basal thyroglobulin levels and tumor standardized uptake value-maximum (SUV-max) on fluorodeoxyglucose positron emission tomography (p = 0.304).

**Conclusion:**

SLC5A5 gene expression is reduced in radioactive iodine-refractory thyroid carcinoma. Furthermore, the decreased expression status of the SLC5A5 gene before preablative iodine treatment may serve as a predictive indicator of future resistance to RAI therapy.

## Introduction

Thyroid carcinoma (TC) is a neoplasm of the endocrine system that accounts for approximately 1–2% of all solid malignancies. Most (90%) of these tumors differentiate and arise from thyroid follicular cells. Patient prognosis remains favorable as long as the tumor maintains its capacity to respond adequately to radioactive iodine (RAI) ^131^ I therapy.^1^ Iodine plays a critical role in the biosynthesis of thyroid hormones and is actively transported into the follicular cells of the thyroid via the sodium–iodide symporter (NIS) on the plasma membrane. Dyshormonogenic congenital hypothyroidism can occur due to loss-of-function mutations in the solute carrier family 5 member 5 (SLC5A5) gene, which is responsible for encoding the NIS.^2^ The efficacy of radioiodine therapy for TC depends on the process of iodide accumulation in tumor cells, which is facilitated by the NIS. In differentiated thyroid tumors, reduced or absence of NIS transport to the plasma membrane often occurs, leading to reduced effectiveness of radioiodine therapy. In cases of differentiated thyroid cancer (DTC), with an incidence rate of four to five new cases per year per million individuals, the ability to take up RAI is lost due to dedifferentiation.^3^ Consequently, these patients do not respond to RAI therapy. These statistics highlight the urgent need to investigate alternative treatment strategies for RAIR-PTC.

The solute carrier (SLC) genes have evolved into at least 52 families with approximately 400 genes in the human genome. Mutations in the human SLC gene have been associated with various illnesses, including hypothyroidism, glycogen storage disorder, and deafness.^4^ The SLC5A5 gene encodes a protein called NIS. The encoded protein is responsible for iodine absorption in organs such as the thyroid gland.^5^ Perchlorate, a common environmental pollutant, disrupts SLC5A5 activity and causes human diseases such as hypothyroidism. Furthermore, hereditary abnormalities in additional genes that may be necessary for effective NIS expression in thyrocytes have not been associated with iodine transport defects. Although SLC5A5 is essential for iodine metabolism and thyroid control, the association between SLC5A5 and thyroid cancer remains complex and poorly understood. It is crucial for clinicians to identify patients with differentiated papillary thyroid cancer who are likely to become resistant to radioiodine treatment in the future. The objective of the study was to assess the expression of SCL5A5 as a potential marker for predicting future resistance to radioiodine treatment.

## Materials And Methods

This was a retrospective study. The study was conducted by retrospectively examining the data between 2016 and 2022. The study assessed various factors, including age, sex, histopathological subtype, metastatic focus, diameter of the largest metastasis in millimeters, thyroglobulin levels (two weeks after the previous surgery) in nanograms per milliliter, and anti-thyroglobulin levels (two weeks after the previous surgery) in international units per milliliter, tumor volume doubling time categorized as less than one year or more than one year, need for systemic chemotherapy, standardized uptake values of the tumor on fluorodeoxyglucose positron emission tomography/computed tomography (FDG-PET/CT) represented by maximum SUV-max, and SLC5A5 expression levels determined from pathological samples. This study included individuals aged >18 years. The individuals studied were divided into two groups: one included patients diagnosed with iodine-refractory thyroid cancer and the other consisted of patients diagnosed with non-refractory thyroid cancer. A total of 83 individuals were included in this study.

### Patient selection

Although there is no consensus on the definition of RAI-refractory disease, the ETA (European Thyroid Association) has published a guideline evaluating it in four categories. 1. Absence of RAI uptake on scintigraphy: although the disease was observed structurally in this patient group, no RAI uptake was observed on diagnostic whole-body iodine scans. 2. While some lesions show RAI involvement, some lesions lack RAI: patients with multiple and large metastatic lesions in whom other diagnostic methods (FDG-PET, diagnostic CT, etc.) detect lesions other than those observed in whole body iodine scan. 3. Progression despite RAI involvement: these patients showed structural or functional progression despite adequate dosage of RAI treatment. 4. Reaching the maximum therapeutic dose of RAI.^6^ In one study, no further complete remission was observed in patients with metastases after a cumulative dose of 600 mCi. Therefore, it was concluded that RAI treatment exceeding 600 mCi would not be beneficial. Due to adverse treatment-related effects, it is also considered RAI refractory if a high cumulative dosage (>600 mCi) is achieved.^7^

### Gene expression analysis methods

The NucleoSpin total RNA FFPE kit (Macherey-Nagel, 740982.50) was used to generate paraffin-embedded tissue samples, which were then subjected to analysis of the SLC5A5 gene according to the manufacturer's recommended protocol. Initially, tissue samples were purified from paraffin and then isolated. cDNA conversion of the RNA samples was performed using the OneScript Plus cDNA synthesis kit (ABMGOOD, G236). Gene expression reactions were conducted using the synthesized SLC5A5 and GAPDH primer samples, 5XHOT FIREPol EvaGreen qPCR Mix Plus (ROX) from SOLIS BIODYNE (08-24-00001), and StepOnePlus Real-Time PCR system from Applied Biosystems (4376600), according to the manufacturer's guidelines.

### Statistical analysis

Data were subjected to statistical analysis using IBM SPSS software (IBM Corp., 2012). The statistical software used in this study was IBM SPSS Statistics for Windows, version 22.0, developed by IBM Corporation (Armonk, New York, USA). The data were classified according to whether they exhibited parametric or non-parametric characteristics. The normality of the data distributions was assessed using the Shapiro–Wilk test and by examining the skewness and kurtosis values. Additionally, the homogeneity of variance was tested using the Levene test. Data that did not show a normal distribution were subjected to statistical analysis using the Kruskal–Wallis test and the Mann–Whitney U test with Bonferroni correction (p < 0.01). Data are presented as mean ± standard deviation (mean ± SD), and a significance level of p < 0.05 was considered statistically significant.

## Results

**Figure 1.**
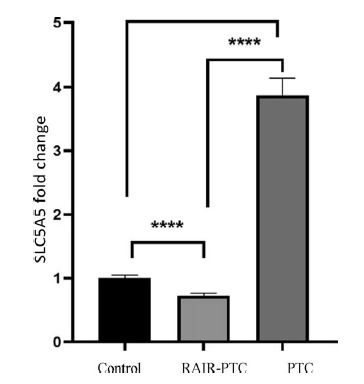
Solute carrier family 5 member 5 (SLC5A5) gene expression in radioactive iodine-refractory thyroid carcinoma. Statistical significance between groups was evaluated by one-way ANOVA and post hoc Tukey test (****p < 0.0001). RAIR-PTC: radioactive iodine-refractory papillary thyroid carcinoma, PTC: papillary thyroid carcinoma.

A total of 83 patients were included in the study. Of these patients, 51 (61.4%) were non-refractory PTC and 32 (38.5%) were RAIR-PTC. In the RAIR-PTC group, 23 patients (71.88%) were female and nine patients (28.13%) were male. In the non-refractory PTC group, 42 (82.35%) were female and nine (17.65%) were male. The gender distribution of the patient and control groups showed no statistically significant difference (p = 0.260, p>0.05). The median ± SD age value in the RAIR-PTC group was 56.56 ± 15.22 years, while in the non-refractory PTC group it was 46.82 ± 12.43. The mean of the RAIR-PTC group was 56.56 ± 15.22, while the median of the non-refractory PTC group was 46.82 ± 12.43. The mean age of diagnosis of the RAIR-PTC group was higher and statistically significant at p = 0.002. Patients were followed up for an average of 8 ± 6.4 years (Table 1). Tall cell subtype was identified as the pathological subtype in 13 specimens from patients with refractory thyroid carcinoma and 11 specimens from patients without refractory thyroid carcinoma. No statistically significant relationship was observed between the refractory thyroid carcinoma and non-refractory groups when comparing the pathological subtype tall cell variant (p = 0.224, p>0.05). No statistically significant correlation was observed between the baseline thyroglobulin levels and the largest metastasis diameter in patients with refractory thyroid carcinoma (p = 0.904, p>0.05) (Table 2). No statistically significant correlation was observed between the basal thyroglobulin levels and the largest SUV-max levels of the tumor on FDG-PET (p = 0.304, p>0.05). There was no difference between the thyroid-stimulating hormone levels of the patients in the groups. The mRNA expression fold change was accepted as control. The fold change was 0.72 in the PTC group and 3.87 in the iodine-refractory thyroid carcinoma group. SLC5A5 gene expression was increased in the PTC group and decreased in the iodine-refractory thyroid carcinoma group. The comparison of the study groups and SLC5A5 gene expression relationship is shown in Figure 1.

## Discussion

Approximately 10% of patients with DTC develop metastases during follow-up. Approximately 33–50% of patients with metastases eventually become resistant to RAI.^8,9^ These patients usually have a poor prognosis. Kersting et al. aimed to evaluate the risk factors that reduced overall survival in patients unresponsive to RAI treatment in 2021 and examined primary tumor size, infiltration of the tissue surrounding the thyroid gland, and tumor volume as risk factors in a total of 51 patients. As a result, primary tumor size >40 mm, extrathyroidal extension, age >55 years, and early thyroglobulin progression (any increase in thyroglobulin levels 4–6 months after initial treatment) were found to be important risk factors for RAI-refractory thyroid carcinoma disease.^10^ It has been reported that older patients are at risk of a higher histological phenotype and a higher RAIR rate. The relationship between age and RAIR-DTC prognosis is unknown. This study found that patients with RAIR-DTC were older than patients with iodine-sensitive thyroid carcinoma.^11^ In the study by Mazzaferri, age was also an important prognostic factor and disease-free survival was longer in younger patients.^12^ The study by Hundahl et al., which included 53,856 patients diagnosed with thyroid cancer, showed that age was one of the important independent prognostic factors in this cancer group. Existing literature does not provide conclusive evidence on the association between gender and RAI refractoriness.^13^ No gender difference in RAI refractoriness was found in this study.

In the study by Faro et al. in 2020, 614 intermediate-risk patients were screened between 1972 and 2015 to compare the effect of RAI treatment and identify risk factors for poor prognosis in intermediate-risk thyroid carcinoma. In that study, risk factors for poor prognosis included the number of affected lymph nodes, the presence of lymph node metastasis, and high serum thyroglobulin levels at the time of ablation.^14^ In the present study, preablative thyroglobulin levels were significantly higher in the radioactive iodine-refractory group. Elevated preablative thyroglobulin levels may serve as an indicator not only of poor prognosis but also of refractoriness to RAI treatment.

The effectiveness of radioiodine therapy depends on the presence of active NIS on the plasma membrane of cells.^15^ Low levels of NIS gene expression are often observed in patients with thyroid cancer, resulting in reduced iodine accumulation. This phenomenon has the potential to induce resistance to RAI therapy in thyroid cancer cells. However, there is a lack of information on the relationship between the NIS expression level in the first total thyroidectomy specimen and the development of RAI refractoriness during the follow-up period. DTC prognosis is influenced by age and older patients having a higher mortality rate. The development process of RAIR is not yet clearly defined. Adequate expression of the sodium–iodine transporter alone is not sufficient. In addition to ideal expression, the realization of appropriate post-translational modifications, the successful integration of these proteins into the plasma membrane, and the successful transport to the plasma membrane are also important variables.^16^ This study focused on the expression level variants of this complex mechanism.

Among other reasons for the decrease in the NIS protein are an increase in some post-translational modifications, dysfunction of clathrin-coated vesicles with overexpression of pituitary tumor transforming gene 1 (PTTG1) and the PTTG-1 binding factor, impairment of NIS glocalization and reaching the surface with activation of the PI3K–AKT pathway, and, more recently, a decrease in ribosomal subunits.^17,18^ Factors affecting the expression of the SLC5A5 gene may have resulted in the deficiency of the NIS and may have resulted in a decrease in NIS expression in the RAIR-PTC group. The limitations of our study include the fact that we did not use immunohistochemical staining or Western blot techniques, and did not have the opportunity to examine all of the aforementioned variables at the same time.

## Conclusion

SLC5A5 gene expression is reduced in radioactive iodine-refractory thyroid carcinoma. Changes in SLC5A5 gene expression may be informative to better understand the pathophysiological mechanism of RAI-refractory disease. Furthermore, it should be taken into account that the efficacy of RAI treatment depends not only on the role of the SLC5A5 gene, but also on the action of other molecules that regulate intracellular metabolism and is mediated by the interaction of all transcriptional and translational pathways.

### Competing interests

The authors have no conflicts of interest to declare.

### Ethics approval

This research was conducted in accordance with the Declaration of Helsinki. Approval was obtained from Gaziantep University Ethics Committee (no. 2021/257).

### Authors' contribution

**ZAS**, **EA**, **AE**, and **SOB** analyzed the data. **ZAS**, **AE**, **SOB**, and **SE** collected the data. **ZAS** wrote the manuscript. **ZAS** and **EA** supervised the article preparation.

## Figures and Tables

**Table 1:** Clinical and demographic parameters of the study groups.

		**Refractory thyroid carcinoma patients (** ***n*** ** = 32)**		**Non-refractory thyroid carcinoma patients (** ***n*** ** = 51)**		

**Variables**		***n***	**%**	***n***	**%**	***p***

Gender	Male	9	28.13	9	17.65	0.260

	Female	23	71.88	42	82.35	

Tumor type	PTC	29	90.6	51	100	0.344

	FTC	3	9.4	NA	NA	

Thyroglobulin doubling time	< 1 year	25	78.1	NA	NA	NA

	>1 year	7	21.9	NA	NA	

Tyrosine kinase inhibitor usage	Yes	13	40.6	NA	NA	NA

	None	19	59.4	NA	NA	


PTC: papillary thyroid carcinoma, FTC: follicular thyroid carcinoma, NA: not applicable.

p < 0.05 is significant.

**Table 2:** Laboratory and disease characteristics of the study groups.

	**Refractory thyroid carcinoma (** ***n*** ** = 32)**		**Non-refractory thyroid carcinoma (** ***n*** ** = 51)**		

**Variables**	**Mean ± SD**	**Median (Q1–Q3)**	**Mean ± SD**	**Median (Q1–Q3)**	***p***

Diagnosis age (years)	56.56 ± 15.22	55 (46.5–70)	46.82 ± 12.43	48 (38–55)	0.002*

Thyroglobulin level after total thyroidectomy (ng/ml)	3588.11 ± 8975.48	278 (65.65–724)	310.46 ± 1796.86	3.21 (0.66–23.7)	0.001*

Anti-thyroglobulin level after total thyroidectomy (ng/ml)	34.11 ± 97.08	0.9 (0.4–7.71)	34.25 ± 153.11	0.9 (0.9–2.7)	0.886

TSH levels before surgery (mIU/L)	1.45 ± 0.9	N/A	1.58 ± 1.1	N/A	0.119


SD: standard deviation, TSH: thyroid-stimulating hormone.

*p < 0.05.
